# Racial and ethnic disparities in diagnosis, management and outcomes of aortic stenosis in the Medicare population

**DOI:** 10.1371/journal.pone.0281811

**Published:** 2023-04-10

**Authors:** Yunus Ahmed, Pieter A. J. van Bakel, Hechuan Hou, Devraj Sukul, Donald S. Likosky, Joost A. van Herwaarden, Daphne C. Watkins, Gorav Ailawadi, Himanshu J. Patel, Michael P. Thompson, for the Structural Heart and Aortic Diseases Outcomes Research Workgroup Investigators

**Affiliations:** 1 Department of Cardiac Surgery, University of Michigan, Ann Arbor, Michigan, United States of America; 2 Department of Vascular Surgery, University Medical Center Utrecht, Utrecht, The Netherlands; 3 Department of Cardiology, University of Michigan, Ann Arbor, Michigan, United States of America; 4 School of Social Work, University of Michigan, Ann Arbor, Michigan, United States of America; Baylor Scott and White, Texas A&M College of Medicine, UNITED STATES

## Abstract

**Importance:**

Aortic stenosis (AS) is one of the most common heart valve conditions and its incidence and prevalence increases with age. With the introduction of transcatheter aortic valve replacement (TAVR), racial and ethnic disparities in AS diagnosis, treatment and outcomes is poorly understood.

**Objective:**

In this study we assessed racial and ethnic disparities in AS diagnosis, treatment, and outcomes among Medicare beneficiaries.

**Design:**

We conducted a population-based cohort study of inpatient, outpatient, and professional claims from a 20% sample of Medicare beneficiaries

**Main outcomes and measures:**

Incidence and Prevalence was determined among Medicare Beneficiaries. Outcomes in this study included management; the number of (non)-interventional cardiology and cardiothoracic surgery evaluation and management (E&M) visits, and number of transthoracic echocardiograms (TTE) performed. Treatment, which was defined as Surgical Aortic Valve Replacement and Transthoracic Aortic Valve Replacement. And outcomes described as All-cause Hospitalizations, Heart Failure Hospitalization and 1-year mortality.

**Results:**

A total of 1,513,455 Medicare beneficiaries were diagnosed with AS (91.3% White, 4.5% Black, 1.1% Hispanic, 3.1% Asian and North American Native) between 2010 and 2018. Annual prevalence of AS diagnosis was lower for racial and ethnic minorities compared with White patients, with adjusted rate ratios of 0.66 (95% CI 0.65 to 0.68) for Black patients, 0.67 (95% CI 0.64 to 0.70) for Hispanic patients and 0.75 (95% CI 0.73 to 0.77) for Asian and North American Native patients as recent as 2018. After adjusting for age, sex and comorbidities, cardiothoracic surgery E&M visits and treatment rates were significantly lower for Black, Hispanic and Asian and North American Native patients compared with White patients. All-cause hospitalization rate was higher for Black and Hispanic patients compared with White patient. 1-year mortality was higher for Black patients, while Hispanic and Asian and North American Native patients had lower 1-year mortality compared with White patients.

**Conclusions and relevance:**

We demonstrated significant racial and ethnic disparities in the diagnosis, management and outcomes of AS. The factors driving the persistence of these disparities in AS care need to be elucidated to develop an equitable health care system.

## Introduction

Aortic stenosis (AS) is one of the most common valvular heart diseases, disproportionately impacting older adults. An estimated 2–4% of patients over the age of 65 in the United States are diagnosed with AS, and its burden on public health and healthcare resources is expected to grow as the population age [1, 2]. Unlike mild to moderate AS, severe AS is associated with reduction in quality of life and life expectancy with medical management. The FDA approval of transcatheter aortic valve replacement (TAVR) in 2011 resulted in a paradigm shift in how severe AS is treated [3–8].

Despite these advances in treatment of AS, disparities in the management and treatment of AS are widely recognized and persistent [9]. Underrepresented minority populations, including Black-Americans, Hispanic-Americans, Asian-Americans and Pacific-Islanders, represent over 30% of the US population, but account for <10% of patients undergoing surgical aortic valve replacement (SAVR) or TAVR [10–12]. These disparities might be reflective of differential prevalence and incidence of AS among racial/ethnic minorities. On the other hand, racial/ethnic disparities in AS may be a result of underdiagnosis due to patient-level factors including patient social network, socioeconomic status, baseline health status, and cultural differences in addition to provider/health system factors including provider availability, hospital quality, and provider quality [13, 14]. These factors may lead to lower community stakeholder engagement and ultimately poor treatment access and quality outcomes for racial/ethnic minorities with AS. Among those patients diagnosed with AS, prior studies have found lower rates of specialist referral and interventions in racial/ethnic minorities, but comparable short- and long term outcomes [10, 15–19]. Establishing equitable outcomes in AS requires a better understanding of the current racial/ethnic disparities in the diagnosis and management of AS in the US. While racial disparities in AS care have been reported, they have not been investigated on a large scale, nor have the utilization of services based on the number of evaluation & management (E&M) visits.

We hypothesized that racial/ethnic disparities exist in AS diagnosis and care. To test our hypothesis, we evaluated the incidence and prevalence of AS across racial/ethnic categories in Medicare beneficiaries, and racial/ethnic differences in the management, treatment, and outcomes after AS diagnosis.

## Methods

### Data sources

We obtained a nationally representative 20% sample of Medicare beneficiaries (n = 16,525,400) from the US Centers for Medicare & Medicaid Services, including the Medicare beneficiary summary, inpatient, outpatient, and carrier files from January 1, 2008 to December 31, 2019. The inpatient files contain institutional claims for inpatient services covered under Medicare Part A. Outpatient files contain institutional claims for outpatient services provided under Medicare Part B. Carrier files contain claims submitted by individual physicians. All files include service dates and *International Classification of Diseases*, *Ninth Revision (ICD-9)* or *International Classification of Diseases*, *Tenth Revision (ICD-10)* diagnosis and procedure codes. This study was deemed exempt from review by the University of Michigan Institutional Review Board.

### Study population

We selected Medicare fee-for-service (FFS) beneficiaries in both Part A and Part B, with at least 2 years of enrollment prior to the index year and 1 year of enrollment after the index year or until death occurred. The summary file contains beneficiary identifiers and demographic information, including age, sex, race, and date of death. Only Medicare beneficiaries aged ≥65 years were included, excluding all patients turning 65 at the start of the study period.

### Diagnosis of AS

We identified beneficiaries with AS using *ICD-9* or *ICD-10* codes (**S1 Table**). AS incidence was determined by identifying beneficiaries with an *ICD-9* or *ICD-10* diagnosis code on 1) a single inpatient claim or 2) one outpatient claim followed by an outpatient/inpatient claim within a period of 365 days. Date of diagnosis was defined by earliest discharge date for a qualifying inpatient claim or the earliest service date of the second qualifying outpatient claim. Two outpatient claims were required to minimize the impact of a ‘rule-out diagnosis’ and to improve the specificity of the chosen AS definition. Beneficiaries were considered an incident case if the AS diagnosis occurred after a minimum 2-year enrollment period without AS diagnosis prior to the index year. Annual incidence was defined as the number of beneficiaries with incident AS during the index year, divided by the number of all beneficiaries at risk during the index year and incidence rate was reported per 1,000 person-years.

AS prevalence was determined using similar criteria as described above during the index year, and only included beneficiaries who were enrolled in Medicare FFS for the entire calendar year. Annual prevalence, calculated on December 31 of each year, was defined as the number of alive beneficiaries with an AS diagnosis divided by the total number of alive beneficiaries during that year. Prevalence rates were reported per 1,000 beneficiaries.

### Outcomes

Among beneficiaries who were diagnosed with AS, we evaluated several outcomes in this study related to AS management and outcomes (**S1 Table)**. Management of AS was evaluated by assessing the number of cardiology, interventional cardiology and cardiothoracic surgery E&M visits, and number of transthoracic echocardiograms (TTE) performed. Procedural interventions (i.e., SAVR and TAVR) were assessed within 1 year of the AS diagnosis. Outcomes of AS management were described as the number of all-cause hospitalizations, heart-failure (HF) hospitalizations and mortality within 1 year of the AS diagnosis.

### Beneficiary information

We evaluated racial and ethnic disparities in AS using the race/ethnicity field in the summary file [20]. This field was used to categorize beneficiaries as White, Black, Hispanic, Asian, and North American Native. Race/ethnicity was self-reported by beneficiaries and acquired from Social Security Administration data. Due to low sample size, Asian and North American Natives were grouped together for analysis.

Beneficiary comorbidity status was described using the Charlson Comorbidity Index, an algorithm consisting of a weighted score of 17 comorbidities based on the risk of 1-year mortality, which has been validated for *ICD-9 and ICD-10* [21, 22]. Comorbidities were identified in the physician claims files (including both inpatient and outpatient visits) up to 12 months prior to the date of diagnosis.

### Statistical analysis

We compared patients’ characteristics in each of 7 years, using chi-square tests for categorical variables. We calculated age- and sex- adjusted AS prevalence and incidence by race and year group. Poisson regression models were performed to test trends of prevalence and incidence over years and to examine the risks of prevalence and incidence for race groups. We also performed Poisson regression models individually to test trends over years by race group and to provide incidence rate ratios (IRR) for cardiology E&M visits, interventional cardiology E&M visits, cardiothoracic surgery E&M visits, TTE, SAVR, TAVR, all-case hospitalization, heart failure (HF) hospitalization, and 1-year mortality. All statistical analyses were conducted using SAS 9.4 software (SAS Institute, Cary, North Caroline, US).

## Results

### Diagnosis

**Fig 1:**
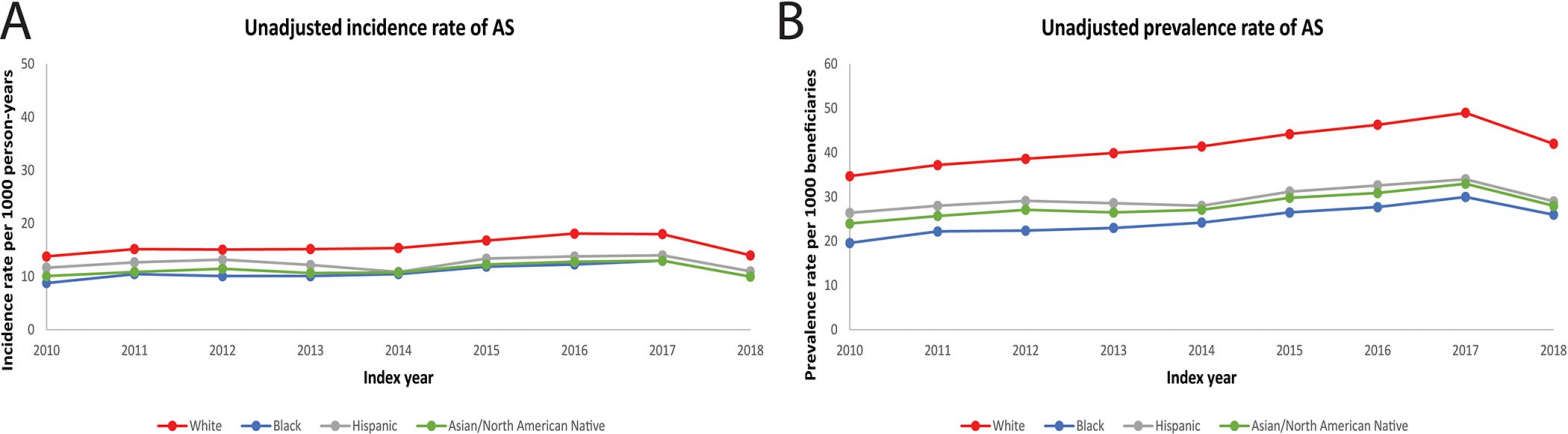
Temporal trends for unadjusted incidence (A) and prevalence (B) rates of aortic stenosis (AS) among Medicare beneficiaries between 2010 and 2018. All p-trends <0.0001.

A total of 1,513,455 patients diagnosed with AS (91.3% White, 4.5% Black, 1.1% Hispanic, 3.1% Asian and North American Native) were identified in the Medicare database between 2010 and 2018 (**Table 1**). Beneficiary demographics by year were reported in **S2 Table**. The AS incidence rate for the overall group increased from 13.5 to 17.0 per 1,000 beneficiaries between 2010 and 2018 (p<0.001, **Fig 1**). Racial/ethnic minorities had significantly lower incidence rates compared with White beneficiaries throughout the study period. The AS prevalence rate for the overall group increased from 33.5 to 40.0 per 1,000 beneficiaries between 2010 and 2018, (p<0.001). Again, racial/ethnic minorities had lower prevalence rates compared with White beneficiaries throughout the study period (p<0.001, **Fig 1**). Disparities in AS prevalence persisted after adjusting for age and sex; adjusted incidence rate ratios, aIRRs (95% CI), varied between 0.62 (0.60, 0.63) to 0.66 (0.65, 0.68) for Black beneficiaries, 0.71 (0.67, 0.74) to 0.67 (0.64, 0.70) for Hispanic beneficiaries and 0.73 (0.71, 0.76) to 0.75 (0.73, 0.77) for Asian and North American Native beneficiaries (all p<0.001). Annual unadjusted IRR for AS incidence and prevalence are reported in **S3 and S4 Tables**.

**Table 1:** Demographics and comorbidities of beneficiaries diagnosed with aortic valve stenosis for the overall sample and stratified by race/ethnicity.

	Overall	White	Black	Hispanic	Asian and North American Native	p-value
**Beneficiaries, No. (%)**	1,513,455 (100)	1,382,263 (91.3)	67,750 (4.5)	16,324 (1.1)	47,118 (3.1)	
**Age**						
66–74, No. (%)	367,440 (24.3)	326,889 (23.7)	21,357 (31.5)	3,356 (20.6)	15,838 (33.6)	< .0001
75–84, No. (%)	660,104 (43.6)	605,316 (43.8)	28,820 (42.5)	6,313 (38.7)	19,655 (41.7)	
85+, No. (%)	485,911 (32.1)	450,058 (32.6)	17,573 (25.9)	6,655 (40.8)	11,625 (24.7)	
**Sex**						
Female, No. (%)	783,707 (51.8)	707,701 (51.2)	42,747 (63.1)	9,828 (60.2)	23,431 (49.7)	< .0001
Male, No. (%)	729,748 (48.2)	674,562 (48.8)	25,003 (36.9)	6,496 (39.8)	23,687 (50.3)	
**Comorbidities**						
CCI 0, No. (%)	292,571 (19.3)	272,125 (19.7)	9,246 (13.7)	2,088 (12.8)	9,112 (19.3)	< .0001
CCI 1–2, No. (%)	564,153 (37.3)	521,170 (37.7)	20,740 (30.6)	5,122 (31.4)	17,121 (36.3)	
CCI 3–4, No. (%)	365,855 (24.2)	333,017 (24.1)	17,400 (25.7)	4,316 (26.4)	11,122 (23.6)	
CCI 5+, No. (%)	290,876 (19.2)	255,951 (18.5)	20,364 (30.1)	4,798 (29.4)	9,763 (20.7)	
**Comorbidities associated with AS**						
Myocardial Infarction, No. (%)	95,277 (6.3)	86,909 (6.3)	4,496 (6.6)	1,160 (7.1)	2,712 (5.8)	< .0001
Congestive Heart Failure, No. (%)	401,377 (26.5)	361,873 (26.2)	22,820 (33.7)	5,529 (33.9)	11,155 (23.7)	< .0001
Peripheral Vascular Disease, No. (%)	432,617 (28.6)	393,193 (28.5)	21,458 (31.7)	5,531 (33.9)	12,435 (26.4)	< .0001
Cerebrovascular Disease, No. (%)	314,675 (20.8)	288,660 (20.9)	13,876 (20.5)	3,669 (22.5)	8,470 (18)	< .0001
Renal Disease, No. (%)	281,300 (18.6)	246,628 (17.8)	20,722 (30.6)	4,051 (24.8)	9,899 (21)	< .0001
Diabetes without complications, No. (%)	485,957 (32.1)	426,946 (30.9)	30,980 (45.7)	8,238 (50.5)	19,793 (42)	< .0001
Diabetes with complications, No. (%)	198,825 (13.1)	171,983 (12.4)	15,142 (22.4)	3,878 (23.8)	7,822 (16.6)	< .0001

AS: Aortic Stenosis, CCI: Charlson Comorbidity Index

### Management

**Fig 2:**
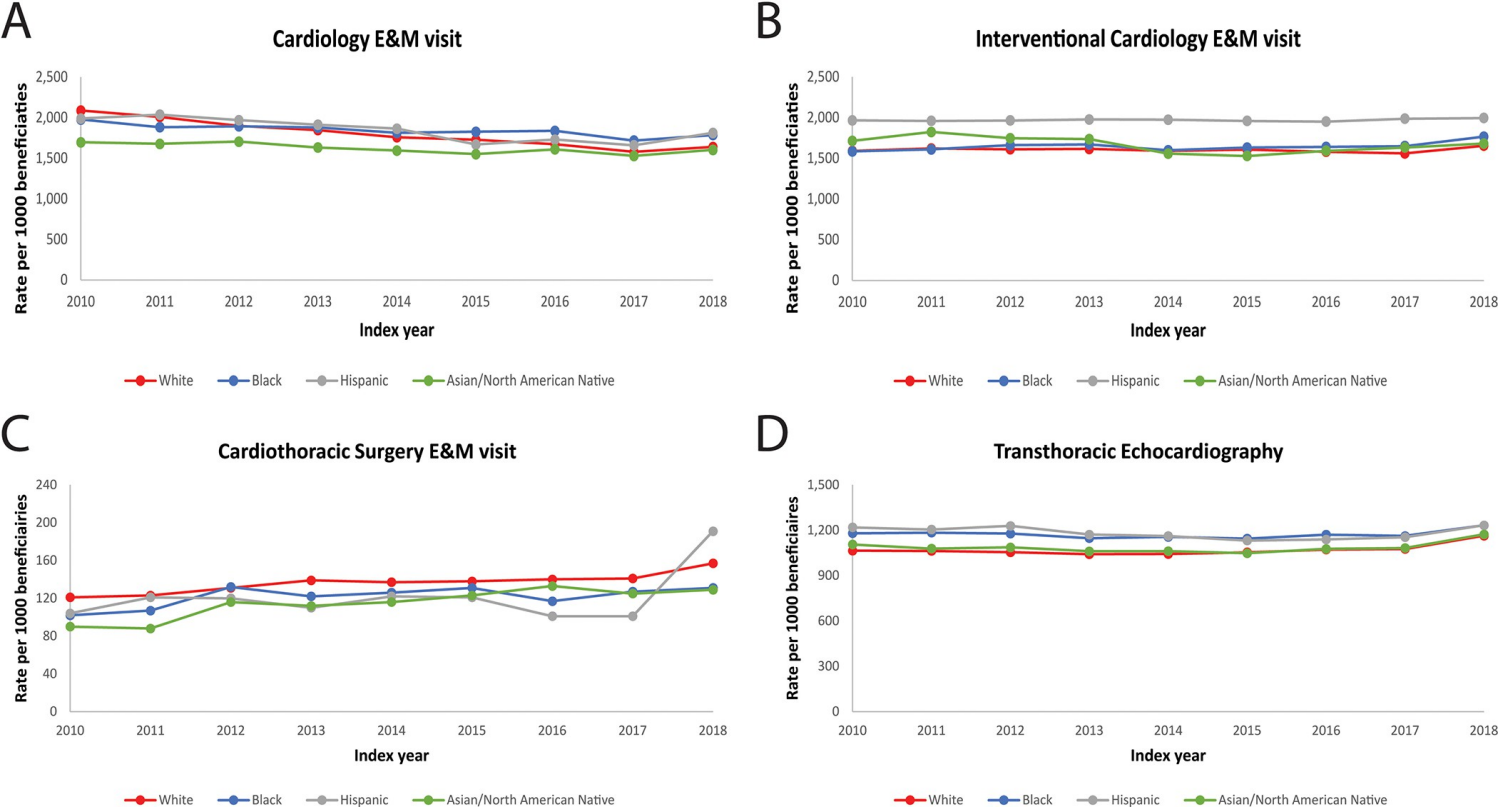
Trends in management of AS (cardiology E&M visits, interventional cardiology E&M visits, cardiothoracic surgery E&M visits, transthoracic echocardiography) E&M: Evaluation and management, all p-trends <0.0001 for cardiology E&M, cardiothoracic surgery E&M and transthoracic echocardiography. Interventional Cardiology not significant.

Trends in the management of AS across racial/ethnic groups can be found in **Fig 2 and S5 Table.** Among beneficiaries who were diagnosed with AS, the rate of cardiology E&M visits were similar for Black and Hispanic patients compared to White patients, while Asian and North American Native patients had significantly lower rates of cardiology E&M visits (**Table 2**). White patients had lower rates of interventional cardiology E&M visits per 1,000 beneficiaries per year compared with racial/ethnic minorities. Interventional cardiology E&M visits remained significantly higher for Hispanic patients compared with White patients (aIRR = 1.18, 1.14 to 1.23; p<0.001), while Black and Asian and North American Native patients had similar rates compared with White patients. White patients also had significantly higher cardiothoracic surgery E&M visits per 1,000 beneficiaries per year compared with all racial/ethnic minority groups (p<0.001), even after adjustment. Rates of TTE use per 1000 beneficiaries were higher for Black and Hispanic patients relative to white patients, while Asian and North American Native patients had similar rates compared with White patients.

**Table 2:** Observed and adjusted racial/ethnic differences in management of aortic valve stenosis (cardiology E&M visit, interventional cardiology E&M visit, cardiothoracic surgery E&M visit, TTE).

	Observed (unadjusted)	p-value	Predicted Mean (95% C.I.)	aIRR (95% C.I.)	p-value
**Cardiology E&M visit**					
White *(per 1K)*	1,787	ref	1804 (1796, 1812)	ref	ref
Black *(per 1K)*	1,839	0.0014	1823 (1787, 1860)	1.01 (0.99, 1.03)	0.30
Hispanic *(per 1K)*	1,849	0.0573	1788 (1718, 1861)	0.99 (0.95, 1.03)	0.66
Asian and North American Native *(per 1K)*	1,611	< .0001	1639 (1596, 1683)	0.91 (0.88, 0.93)	< .0001
**Interventional Cardiology E&M visit**					
White *(per 1K)*	1,602	ref	1598 (1591, 1606)	ref	ref
Black *(per 1K)*	1,647	0.0011	1601 (1570, 1632)	1.00 (0.98, 1.02)	0.9
Hispanic *(per 1K)*	1,970	< .0001	1891 (1820, 1966)	1.18 (1.14, 1.23)	< .0001
Asian and North American Native *(per 1K)*	1,657	< .0001	1624 (1585, 1663)	1.02 (0.99, 1.04)	0.21
**Cardiothoracic Surgery E&M visit**					
White *(per 1K)*	137	ref	128 (127, 129)	ref	ref
Black *(per 1K)*	122	< .0001	109 (104, 115)	0.85 (0.81, 0.90)	< .0001
Hispanic *(per 1K)*	121	< .0001	111 (101, 121)	0.87 (0.79, 0.95)	0.002
Asian and North American Native *(per 1K)*	118	< .0001	105 (98, 113)	0.82 (0.76, 0.88)	< .0001
**TTE**					
White *(per 1K)*	1,071	ref	1069 (1067, 1071)	ref	ref
Black *(per 1K)*	1,173	< .0001	1152 (1142, 1162)	1.08 (1.07, 1.09)	< .0001
Hispanic *(per 1K)*	1,183	< .0001	1175 (1154, 1196)	1.10 (1.08, 1.12)	< .0001
Asian and North American Native *(per 1K)*	1,089	0.0003	1076 (1064, 1088)	1.01 (0.99, 1.02)	0.28

E&M: Evaluation & management, TTE: transthoracic echocardiography, aIRR: adjusted incident rate ratio

### Treatment

**Fig 3:**
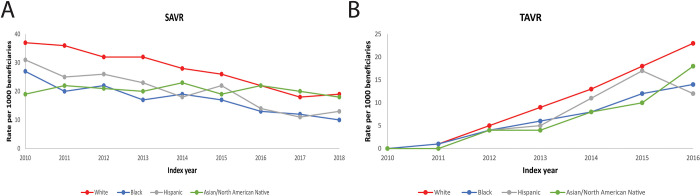
Trends in treatment of AS (SAVR, TAVR) SAVR: Surgical aortic valve replacement, TAVR: Transcatheter aortic valve replacement. All p-trends <0.0001.

Annual rates of SAVR significantly decreased over time and rates of TAVR rates significantly increased over time across all racial/ethnic groups (**Fig 3 and S6 Table**). Crude rates of SAVR were higher for White patients compared with all racial/ethnic minority groups (**Table 3**). After adjusting for age, sex and comorbidities, rates of SAVR remained significantly lower for racial/ethnic minority patients relative to White beneficiaries. Similarly, crude rates of TAVR were higher for White patients relative to racial/ethnic minority beneficiaries, which persisted after adjustment.

**Table 3:** Observed and adjusted racial/ethnic differences in the treatment of aortic stenosis (SAVR, TAVR).

	Observed (unadjusted)	p-value	Predicted Mean (95% C.I)	aIRR (95% C.I.)	p-value
**SAVR**					
White *(per 1K)*	27	ref	26.3 (26.1, 27)	ref	ref
Black *(per 1K)*	17	< .0001	16 (15, 17)	0.62 (0.59, 0.66)	< .0001
Hispanic *(per 1K)*	20	< .0001	22 (20, 25)	0.85 (0.76, 0.95)	0.0051
Asian and North American Native *(per 1K)*	20	< .0001	17 (16, 18)	0.64 (0.59, 0.69)	< .0001
**TAVR (2012–2018)**					
White *(per 1K)*	16	ref	11.0 (10.8, 11.3)	ref	ref
Black *(per 1K)*	10	< .0001	7 (6, 8)	0.64 (0.58, 0.72)	< .0001
Hispanic *(per 1K)*	11	< .001	7 (6, 8)	0.62 (0.51, 0.77)	< .0001
Asian and North American Native *(per 1K)*	11	< .0001	8 (7, 9)	0.71 (0.63, 0.8)	< .0001

SAVR: surgical aortic valve replacement, TAVR: transcatheter aortic valve replacement, aIRR: adjusted incident rate ratio

(Un)adjusted TAVR rates were calculated beginning in 2012 to account for the first full year of TAVR.

### Outcomes

**Fig 4:**
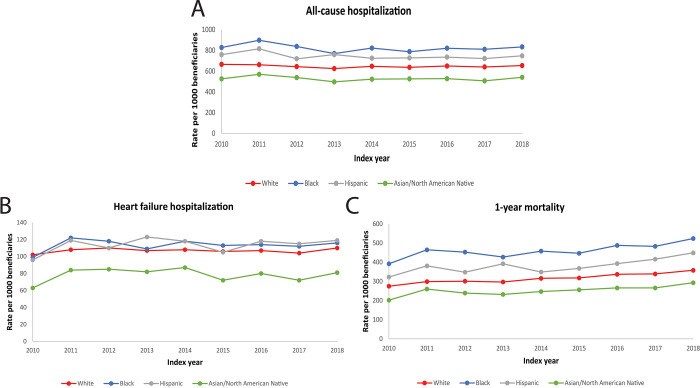
Trends AS outcomes (all-cause hospitalization, heart failure hospitalization, 1-year mortality) p-trends <0.0001 for all-cause hospitalization and heart failure hospitalization. 1-year mortality not significant.

Crude rates of all-cause hospitalizations and HF hospitalizations increased for all racial/ethnic groups during the study period (all p<0.001), while 1-year mortality rates had no significant trends (**Fig 4 and S7 Table**). After adjusting for age, sex and comorbidities, Black patients had higher rates of all-cause hospitalization and HF hospitalizations relative to White patients (**Table 4**). Asian and North American Native patients had significantly lower rates of all-cause and HF hospitalizations compared with White patients. There was no difference in all-cause or HF hospitalization rates between White and Hispanic patients. Black and Hispanic patients had a higher crude 1-year mortality relative to White patients. After adjusting for age, sex and comorbidities, 1-year mortality was higher for Black patients, while Hispanic and Asian and North American Native patients had lower rates of 1-year mortality.

**Table 4:** Observed and adjusted racial/ethnic differences in the outcomes of beneficiaries with aortic stenosis (all-cause hospitalization, heart failure hospitalization, 1-year mortality).

	Observed (unadjusted)	p-value	Predicted Mean (95% C.I.)	IRR (95% C.I.)	p-value
**All-cause hospitalization**					
White *(per 1K)*	647	ref	633 (631, 635)	ref	ref
Black *(per 1K)*	822	< .0001	751 (740, 763)	1.19 (1.17, 1.21)	< .0001
Hispanic *(per 1K)*	746	< .0001	650 (631, 670)	1.03 (1.00, 1.06)	0.09
Asian and North American Native *(per 1K)*	527	< .0001	525 (514, 537)	0.83 (0.81, 0.85)	< .0001
**Heart failure hospitalization**					
White *(per 1K)*	318	ref	293 (291, 295)	ref	ref
Black *(per 1K)*	462	< .0001	386 (377, 394)	1.32 (1.29, 1.35)	< .0001
Hispanic *(per 1K)*	380	< .0001	295 (283, 309)	1.01 (0.96, 1.05)	0.73
Asian and North American Native *(per 1K)*	256	< .0001	240 (233, 248)	0.82 (0.79, 0.85)	< .0001
**1-year mortality**					
White *(per 1K)*	107	ref	95 (95, 96)	ref	ref
Black *(per 1K)*	114	< .0001	101 (98, 103)	1.06 (1.03, 1.08)	< .0001
Hispanic *(per 1K)*	114	0.004	80 (76, 84)	0.84 (0.8, 0.88)	< .0001
Asian and North American Native *(per 1K)*	78	< .0001	75 (72, 77)	0.78 (0.76, 0.81)	< .0001

## Discussion

In this study we explored the role of race and ethnicity in AS using a population-based sample of Medicare beneficiaries in the United States. We found that racial and ethnic minorities were associated with significantly lower rates of incident and prevalent AS diagnoses compared with White beneficiaries. Once diagnosed with AS, cardiologist evaluations were comparable across all racial and ethnic groups, except for Asian and North American Native. In contrast, both evaluation by a cardiothoracic surgeon and treatment rates were markedly lower for minority populations compared with White patients. In addition, outcomes of all-cause hospitalizations, HF hospitalizations and 1-year mortality were worse for Black patients compared with White patients. Collectively, these findings suggest that racial and ethnic disparities are present across the continuum of AS diagnosis, management, and outcomes.

Prior reports evaluating disparities in AS care have predominantly focused on outcomes conditional on receiving treatment, and thereby neglected the role of access to healthcare [10, 11, 16–19]. These studies, using in-hospital data, have reported a lower incidence and prevalence of AS among racial/ethnic minorities, although these demographic groups are associated with higher rates of AS-related risk factors, including obesity, hypertension, diabetes mellitus, and chronic kidney disease [11, 15, 19]. Our study confirms these findings among a broad population-based sample. Compared with racial/ethnic distribution among all Medicare beneficiaries, we report a lower share of AS diagnosis among racial/ethnic minorities (9% vs 5% Black, 6% vs 1% Hispanic), while White beneficiaries accounted for a higher share (91% vs 81%). While these findings might allude to racial/ethnic disparities in AS diagnosis, we cannot accurately affirm this without systematic echocardiography screening to determine presence of AS and specify disease severity. There is a need for better understanding of epidemiology of AS in a broad population. Some studies suggest that racial/ethnic minorities might be a lower risk of developing severe AS. As previously reported by Patel and colleagues [15], lower prevalence of AS among racial minorities could indeed be real, and not based of disparity in diagnosis. Unfortunately, we are not able to ascertain this using out current claims-based data, and therefore disparities in AS diagnosis are not sustained. A study by Budoff and colleagues [23] reports significantly lower prevalence of coronary artery calcification among Black and Hispanic patients compared with White patients. As coronary artery disease is strongly related to AS, this might further support our finding of lower AS prevalence among racial/ethnic minorities. Etiologic studies of AS have suggested differences in bicuspid aortopathy or genetic factors across demographic groups, which may result in differences in AS incidence and prevalence [10]. However, assuming race is a social construct, this does not provide an explanation for differential prevalence of AS. While not sustained by our current findings, racial/ethnic disparities in AS incidence and prevalence could also be explained by non-physiologic factors, such as access to and quality of care for minority populations [24].

Once diagnosed with AS, patients of all demographic groups appear to have similar rates of cardiologist evaluation. A recent study found that among patients with severe symptomatic AS, Black patients had lower rates of identifiable cardiology visits and worse outcomes [25]. In order to fully assess adequacy of the evaluation, we must consider confounding factors, including access to care, quality of care, implicit bias, socioeconomic factors and etiology. Interestingly, and in contrast to what was expected, TTE surveillance was found to be higher among racial/ethnic minorities. While this finding seems conflicting, it could be the result of the differential disease severity (i.e., higher prevalence of severe AS) among racial minorities, prompting more frequent surveillance. Finally, our study validates existing literature highlighting racial and ethnic disparities in evaluation by a cardiothoracic surgeon [18, 26]. This finding may be the result of differences in referral patterns, availability of specialists and hospitals that can perform AVR in the region or patient-related factors (e.g., refusal of intervention, socioeconomic and cultural factors restricting access to care).

Procedural intervention rates among patients diagnosed with AS were significantly lower for racial and ethnic minorities. Prior studies have also suggested this finding [16, 17, 27]. Bach et al. found that one third of patients with severe AS do not undergo AVR, despite being symptomatic [28]. These disparities may in part be explained by differences in subspecialty evaluation and referral patterns. With the advent of a new minimally invasive therapy such as TAVR, it is important to address the disparities in treatment as they could be most beneficial in those patients with high-risk factors.

Across all racial and ethnic categories in our study, Black AS patients were associated with the highest rates of all-cause hospitalizations, HF hospitalizations, and 1-year mortality. Prior studies have found similar racial/ethnic differences in outcomes for chronic cardiovascular diseases (e.g. heart failure, and coronary artery disease) [29, 30]. This finding is most likely attributable to both a higher risk profile, including poor nutrition and living conditions, the potential progressive state of disease and limited access to adequate intervention [31, 32]. Societal factors and social determinants of health, including income, housing and education might also influence outcomes in this group [33, 34]. However, Hispanic, Asian, and North American Native patients in our study appear to have similar high-risk profiles and limited access to care, actually showed equal or even better outcomes compared with White patients. Previous studies found either similar or worse short- and long term outcomes among racial/ethnic minorities following AS diagnosis [10, 17, 25, 27]. It is evident that in order to address disparities in outcomes following AS, we must strive to ensure adequate, equal and timely management and treatment across all racial and ethnic populations. Future research should focus on exploring potential confounding factors that may further explain our findings, including geographical variations, population-based supply of providers and services, social determinants of health, provider implicit bias and socioeconomic status.

There are several limitations to this study. First, current Medicare practices combine race and ethnicity as well as racial groups, limiting accurate classification of the population, where many people identify as belonging to more than one group [20]. Second, while this study is focused on all Medicare FFS beneficiaries with AS, claims data are limited with respect to clinical characteristics. Thus, we were not able to confirm the severity or progression of disease at diagnosis, which may explain some of the racial and ethnic disparities observed in our study. Third, in this study Medicare beneficiaries with AS were identified based on ICD-9/ICD-10 codes. Coding practices have changed over our study period (i.e., transitioning from ICD-9 to ICD-10 coding), which could have potentially affected our estimates. However, the sensitivity analyses using different definitions of AS did not appear to qualitatively impact our estimates of incident and prevalent AS cases. Fourth, our study is limited by the inability to include non-FFS Medicare patients, especially given greater representation of racial and ethnic minorities in Medicare Advantage. Finally, our study outcomes were limited to hospitalizations and mortality, and does not evaluate other potential important outcomes such as health care expenditures and quality of life.

## Conclusion

In conclusion, we demonstrated that racial and ethnic minorities were significantly less frequently diagnosed with AS and had disparities in both adequate management and outcomes in a population-based cohort of Medicare beneficiaries. While the etiology of these findings remains unclear, the driving factors leading to these disparities will need further evaluation to address this pathology equitably across demographic groups.

## Supporting information

S1 Table Definitions used for management, treatment, and outcomes of AS. (DOCX)

S2 Table Beneficiary demographics by year. (DOCX)

S3 Table Unadjusted and adjusted incidence rates and rate ratios (including interaction term race*year). (DOCX)

S4 Table Unadjusted and adjusted prevalence rates and rate ratios (including interaction terms race*year, sensitivity analysis for dual eligibility adjustment). (DOCX)

S5 Table Trends in management of AS. (DOCX)

S6 Table Trends in treatment of AS. (DOCX)

S7 Table Trends in AS outcomes. (DOCX)
