# Supplementary material for: Racial and ethnic disparities in diagnosis, management and outcomes of aortic stenosis in the Medicare population
Source: PLoS One. 2023 Apr 10;18(4):e0281811. doi: 10.1371/journal.pone.0281811 (PMC10085041; doi:10.1371/journal.pone.0281811)
Supplement: S1 Table — (DOCX) [file pone.0281811.s001.docx]

**Table S1:** Definitions used for management, treatment and outcomes of AS

| **Variable** | **Definition** |
| --- | --- |
| Aortic Stenosis diagnosis codes | - ICD-9 diagnosis codes:   **424.1** Aortic valve disorders  **746.3** Congenital stenosis of aortic valve  **395.0** Rheumatic aortic stenosis  **395.2** Rheumatic aortic stenosis with insufficiency  **396.0** Mitral valve stenosis and aortic valve stenosis  **396.2** Mitral valve insufficiency and aortic valve insufficiency  **397.9** Rheumatic diseases of endocardium, valve unspecified   - ICD-10 diagnosis codes   **I35.0** Nonrheumatic aortic valve stenosis  **I35.2** Nonrheumatic aortic valve stenosis  **Q23.0** Congenital stenosis of aortic valve  **I06.0** Rheumatic aortic stenosis  **I06.2** Rheumatic aortic stenosis with insufficiency  **I08.0** Rheumatic disorders of both mitral and aortic valves  **I08.2** Rheumatic disorders of both aortic and tricuspid valves  **I08.3** Combined rheumatic disorders of mitral, aortic and tricuspid valves |
| Cardiology E&M visits | - Provider specialty code ‘’**06**’’ or ‘’**C3**’’ - Has not billed PCI CPT codes |
| Interventional Cardiology E&M visit | - Provider specialty code ‘’**06**’’ or ‘’**C3**’’ - Billed PCI CPT codes at least once:   **PCI CPT 92920** Angioplasty, single vessel  **PCI CPT 92928** Stent, single vessel  **PCI CPT 92933** Atherectomy + stent, single vessel  **PCI CPT 92980** Transcatheter placement of an intracoronary stent(s), percutaneous, with or without other therapeutic intervention, any method: single vessel  **PCI CPT 92982** Percutaneous transluminal coronary balloon angioplasty, single vessel |
| Cardiothoracic surgery E&M visit | - Provider specialty codes ‘’**33**’’ or ‘’**78**’’ |
| TTE | - CPT codes:   **99303** Echo transthoracic  **99304** Echo transthoracic  **99306** TTE w/ Doppler complete  **99307** TTE w/o Doppler complete  **99308** TTE f-up or lmtd |
| SAVR | - ICD-9 procedure codes:   **35.21** Open and other replacement of aortic valve with tissue graft  **35.22** Open and other replacement of aortic valve   - ICD-10 procedure codes:   **02RF07Z** Replacement of Aortic Valve with Autologous Tissue Substitute, Open Approach  **02RF08Z** Replacement of Aortic Valve with Zooplastic Tissue, Open Approach  **02RF0KZ** Replacement of Aortic Valve with Nonautologous Tissue Substitute, Open Approach  **02RF47KZ** Replacement of Aortic Valve with Autologous Tissue Substitute, Percutaneous Endoscopic Approach  **02RF48Z** Replacement of Aortic Valve with Zooplastic Tissue, Percutaneous Endoscopic Approach  **02RF4KZ** Replacement of Aortic Valve with Nonautologous Tissue Substitute, Percutaneous Endoscopic Approach  **02RF0JZ** Replacement of Aortic Valve with Synthetic Substitute, Open Approach  **02RF4JZ** Replacement of Aortic Valve with Synthetic Substitute, Percutaneous Endoscopic Approach  **X2RF032** Replacement of Aortic Valve using Zooplastic Tissue, Rapid Deployment Technique, Open Approach, New Technology Group 2  **X2RF432** Replacement of Aortic Valve using Zooplastic Tissue, Rapid Deployment Technique, Percutaneous Endoscopic Approach, New Technology Group 2 |
| TAVR | - ICD-9 procedure codes:   **35.05** Endovascular replacement of aortic valve  **35.06** Transapical replacement of aortic valve   - ICD-10 procedure codes:   **02RF37Z** Replacement of Aortic Valve with Autologous Tissue Substitute, Percutaneous Approach  **02RF38Z** Replacement of Aortic Valve with Zooplastic Tissue, Percutaneous Approach  **02RF3JZ R**eplacement of Aortic Valve with Synthetic Substitute, Percutaneous Approach  **02RF3KZ** Replacement of Aortic Valve with Nonautologous Tissue Substitute, Percutaneous Approach  **02RF37H** Replacement of Aortic Valve with Synthetic Substitute, Transapical, Percutaneous  **02RF38H** Replacement of Aortic Valve with Zooplastic Tissue, Transapical, Percutaneous Approach  **02RF3JH** Replacement of Aortic Valve with Synthetic Substitute, Transapical, Percutaneous Approach  **02RF3KH** Replacement of Aortic Valve with Nonautologous Tissue Substitute, Transapical, Percutaneous Approach  **X2RF332** Replacement of Aortic Valve using Zooplastic Tissue, Rapid Deployment Technique, Percutaneous Approach, New Technology Group 2 |
| All-cause hospitalizations | If there was a hospitalization for the patient |
| Heart failure hospitalizations | Hospitalizations with the following ICD-9 diagnosis codes:  **402.01** Malignant hypertensive heart disease with heart failure  **402.11** Benign hypertensive heart disease with heart failure  **402.91** Unspecified hypertensive heart disease with heart failure  **404.01** Hypertensive heart and chronic kidney disease, malignant, with heart failure and with chronic kidney disease stage I through stage IV, or unspecified  **404.03** Hypertensive heart and chronic kidney disease, malignant, with heart failure and with chronic kidney disease stage V or end stage renal disease  **404.11** Hypertensive heart and chronic kidney disease, benign, with heart failure and with chronic kidney disease stage I through stage IV, or unspecified  **404.13** Hypertensive heart and chronic kidney disease, benign, with heart failure and chronic kidney disease stage V or end stage renal disease  **404.91** Hypertensive heart and chronic kidney disease, unspecified, with heart failure and with chronic kidney disease stage I through stage IV, or unspecified  **404.93** Hypertensive heart and chronic kidney disease, unspecified, with heart failure and chronic kidney disease stage V or end stage renal disease  **428.xx** Heart failure  Hospitalizations with the following ICD-10 diagnosis codes:  **I11.0** Hypertensive heart disease with heart failure  **I13.0** Hypertensive heart disease with heart failure  **I13.2** Hypertensive heart and chronic kidney disease with heart failure and with stage 5 chronic kidney disease, or end stage renal disease  **I50.xx** Heart failure |
| 1-year mortality | Identified by death date |
