# Supplementary material for: Racial and ethnic disparities in diagnosis, management and outcomes of aortic stenosis in the Medicare population
Source: PLoS One. 2023 Apr 10;18(4):e0281811. doi: 10.1371/journal.pone.0281811 (PMC10085041; doi:10.1371/journal.pone.0281811)
Supplement: S2 Table — (DOCX) [file pone.0281811.s002.docx]

**Table S2:** Patient demographics by year

|  | **2010** | **2011** | **2012** | **2013** | **2014** | **2015** | **2016** | **2017** | **2018** | **p-value** | **2010-2018** |
| --- | --- | --- | --- | --- | --- | --- | --- | --- | --- | --- | --- |
| **Overall** | 139,730 | 151,152 | 155,396 | 160,289 | 165,989 | 176,974 | 188,542 | 199,413 | 75,970 |  | 1,513,455 |
| **Age** |  | | | | | | | | | | |
| 66-74, No. (%) | 32,915 (23.6%) | 35,104 (23.2%) | 36,084 (23.2%) | 37,649 (23.5%) | 39,692 (23.9%) | 43,481 (24.6%) | 47,420 (25.2%) | 50,960 (25.6%) | 44,135 (25.1%) | < .0001 | 367,440 (24.3%) |
| 75-84, No. (%) | 63,823 (45.7%) | 67,679 (44.8%) | 68,943 (44.4%) | 70,394 (43.9%) | 72,103 (43.4%) | 75,913 (42.9%) | 80,284 (42.6%) | 85,314 (42.8%) | 75,651 (43%) |  | 660,104 (43.6%) |
| 85+, No. (%) | 42,992 (30.8%) | 48,369 (32%) | 50,369 (32.4%) | 52,246 (32.6%) | 54,194 (32.7%) | 57,580 (32.5%) | 60,838 (32.3%) | 63,139 (31.7%) | 56,184 (31.9%) |  | 485,911 (32.1%) |
| **Sex** |  | | | | | | | | | | |
| Female, No. (%) | 73,547 (52.6%) | 79,596 (52.7%) | 81,240 (52.3%) | 83,225 (51.9%) | 85,236 (51.4%) | 90,725 (51.3%) | 97,067 (51.5%) | 102,817 (51.6%) | 90,254 (51.3%) | < .0001 | 783,707 (51.8%) |
| Male, No. (%) | 66,183 (47.4%) | 71,556 (47.3%) | 74,156 (47.7%) | 77,064 (48.1%) | 80,753 (48.7%) | 86,249 (48.7%) | 91,475 (48.5%) | 96,596 (48.4%) | 85,716 (48.7%) |  | 729,748 (48.2%) |
| **Comorbidities** |  | | | | | | | | | | |
| CCI 0, No. (%) | 27,820 (19.9%) | 29,143 (19.3%) | 29,670 (19.1%) | 30,490 (19.0%) | 31,119 (18.8%) | 33,030 (18.7%) | 34,963 (18.5%) | 41,564 (20.8%) | 34,772 (19.8%) | < .0001 | 292,571 (19.3%) |
| CCI 1-2, No. (%) | 55,188 (39.5%) | 58,233 (38.5%) | 59,226 (38.1%) | 60,513 (37.8%) | 62,562 (37.7%) | 65,935 (37.3%) | 68,001 (36.1%) | 72,207 (36.2%) | 62,288 (35.4%) |  | 564,153 (37.3%) |
| CCI 3-4, No. (%) | 33,914 (24.3%) | 36,845 (24.4%) | 38,037 (24.5%) | 39,447 (24.6%) | 40,481 (24.4%) | 43,278 (24.5%) | 45,707 (24.2%) | 46,713 (23.4%) | 41,433 (23.6%) |  | 365,855 (24.2%) |
| CCI 5+, No. (%) | 22,808 (16.3%) | 26,931 (17.8%) | 28,463 (18.3%) | 29,839 (18.6%) | 31,827 (19.2%) | 34,731 (19.6%) | 39,871 (21.2%) | 38,929 (19.5%) | 37,477 (21.3%) |  | 290,876 (19.2%) |
| **Comorbidities associated with AS** |  | | | | | | | | | | |
| Myocardial Infarction, No. (%) | 8,225 (5.9%) | 9,354 (6.2%) | 9,578 (6.2%) | 9,726 (6.1%) | 10,115 (6.1%) | 10,892 (6.2%) | 12,102 (6.4%) | 13,066 (6.6%) | 12,219 (6.9%) | < .0001 | 95,277 (6.3%) |
| Congestive Heart Failure, No. (%) | 37,428 (26.8%) | 40,984 (27.1%) | 41,407 (26.7%) | 41,795 (26.1%) | 42,917 (25.9%) | 45,545 (25.7%) | 49,855 (26.4%) | 52,543 (26.4%) | 48,903 (27.8%) | < .0001 | 401,377 (26.5%) |
| Peripheral Vascular Disease, No. (%) | 38,218 (27.4%) | 42,137 (27.9%) | 43,080 (27.7%) | 43,896 (27.4%) | 46,007 (27.7%) | 49,085 (27.7%) | 55,477 (29.4%) | 59,665 (29.9%) | 55,052 (31.3%) | < .0001 | 432,617 (28.6%) |
| Cerebrovascular Disease, No. (%) | 37,066 (26.5%) | 40,525 (26.8%) | 41,770 (26.9%) | 43,256 (27%) | 44,116 (26.6%) | 46,477 (26.3%) | 42,592 (22.6%) | 9,947 (5%) | 8,926 (5.1%) | < .0001 | 314,675 (20.8%) |
| Renal Disease, No. (%) | 18,639 (13.3%) | 22,750 (15.1%) | 24,947 (16.1%) | 27,343 (17.1%) | 30,233 (18.2%) | 33,816 (19.1%) | 39,067 (20.7%) | 43,378 (21.8%) | 41,127 (23.4%) | < .0001 | 281,300 (18.6%) |
| Diabetes without complications, No. (%) | 42,821 (30.7%) | 47,861 (31.7%) | 50,351 (32.4%) | 51,923 (32.4%) | 53,949 (32.5%) | 57,312 (32.4%) | 61,204 (32.5%) | 63,843 (32%) | 56,693 (32.2%) | < .0001 | 485,957 (32.1%) |
| Diabetes with complications, No. (%) | 14,250 (10.2%) | 16,679 (11%) | 18,056 (11.6%) | 19,095 (11.9%) | 20,392 (12.3%) | 22,490 (12.7%) | 27,006 (14.3%) | 31,063 (15.6%) | 29,794 (16.9%) | < .0001 | 198,825 (13.1%) |
| **Race/Ethnicity** |  | | | | | | | | | | |
| White, No. (%) | 128,475 (92%) | 138,409 (91.6%) | 142,205 (91.5%) | 146,826 (91.6%) | 152,171 (91.7%) | 161,795 (91.4%) | 171,975 (91.2%) | 181,000 (90.8%) | 159,407 (90.6%) | < .0001 | 1,382,263 (91.3%) |
| Black, No. (%) | 5,995 (4.3%) | 6,898 (4.6%) | 6,975 (4.5%) | 7,183 (4.5%) | 7,407 (4.5%) | 7,966 (4.5%) | 8,435 (4.5%) | 9,005 (4.5%) | 7,886 (4.5%) |  | 67,750 (4.5%) |
| Hispanic, No. (%) | 1,725 (1.2%) | 1,862 (1.2%) | 1,907 (1.2%) | 1,856 (1.2%) | 1,683 (1%) | 1,736 (1%) | 1,849 (1%) | 1,971 (1%) | 1,735 (1%) |  | 16,324 (1.1%) |
| Asian and North American Native, No. (%) | 3,535 (2.5%) | 3,983 (2.6%) | 4,309 (2.8%) | 4,424 (2.8%) | 4,728 (2.9%) | 5,477 (3.1%) | 6,283 (3.3%) | 7,437 (3.7%) | 6,942 (3.9%) |  | 47,118 (3.1%) |

AS: Aortic Stenosis

CCI: Charlson Comorbidity Index
