# Supplementary material for: Racial and ethnic disparities in diagnosis, management and outcomes of aortic stenosis in the Medicare population
Source: PLoS One. 2023 Apr 10;18(4):e0281811. doi: 10.1371/journal.pone.0281811 (PMC10085041; doi:10.1371/journal.pone.0281811)
Supplement: S3 Table — (DOCX) [file pone.0281811.s003.docx]

**Table S3:** Unadjusted and adjusted incidence rates and rate ratios

| **Characteristic** | **Number of beneficiaries with AS (incidence rate per 1000 beneficiaries)** | | | | | | |  |  |  |  |
| --- | --- | --- | --- | --- | --- | --- | --- | --- | --- | --- | --- |
|  | **2010** | **2011** | **2012** | **2013** | **2014** | **2015** | **2016** | **2017** | **2018** | **p-trend** | **2010-2018** |
| **Overall** | 13.5 | 14.9 | 14.8 | 14.8 | 15.0 | 16.5 | 17.7 | 17.0 | 14.0 | < .0001 | 15.4 |
| **Age** |  | | | | | | | | | | |
| 66-74 | 8.3 | 8.8 | 8.8 | 8.9 | 9.0 | 10.0 | 10.8 | 11.0 | 8.0 | < .0001 | 9.3 |
| 75-84 | 15.1 | 16.5 | 16.6 | 16.5 | 16.9 | 18.6 | 20.0 | 20.0 | 15.0 | < .0001 | 17.2 |
| 85+ | 22.3 | 26.0 | 25.4 | 25.6 | 26.5 | 29.1 | 31.8 | 32.0 | 27.0 | < .0001 | 27.2 |
| **Sex** |  | | | | | | | | | | |
| Female | 12.5 | 13.8 | 13.8 | 13.8 | 13.9 | 15.3 | 16.4 | 16.0 | 13.0 | < .0001 | 14.2 |
| Male | 15.0 | 16.5 | 16.3 | 16.2 | 16.6 | 18.2 | 19.4 | 19.0 | 5.0 | < .0001 | 16.9 |
| **Race/Ethnicity** |  | | | | | | | | | | |
| White | 13.8 | 15.2 | 15.1 | 15.2 | 15.4 | 16.8 | 18.1 | 18.0 | 14.0 | < .0001 | 16.0 |
| Black | 8.8 | 10.5 | 10.1 | 10.1 | 10.5 | 11.9 | 12.3 | 13.0 | 10.0 | < .0001 | 11.0 |
| Hispanic | 11.7 | 12.7 | 13.2 | 12.2 | 10.9 | 13.4 | 13.8 | 14.0 | 11.0 | < .0001 | 12.8 |
| Asian and North American Native | 10.1 | 10.9 | 11.5 | 10.7 | 10.8 | 12.3 | 12.8 | 13.0 | 10.0 | < .0001 | 11.7 |
|  | | | | | | | | | | | |
| **Race/Ethnicity** | **2010** | **2011** | **2012** | **2013** | **2014** | **2015** | **2016** | **2017** | **2018** | **p-trend** | **2010-2016** |
| *White (reference)* | 13.8 | 15.2 | 15.1 | 15.2 | 15.4 | 16.8 | 18.1 | 18.0 | 14.0 | < .0001 | 15.7 |
| *Black* | 8.8 | 10.5 | 10.1 | 10.1 | 10.5 | 11.9 | 12.3 | 12.0 | 10.0 | < .0001 | 10.7 |
| Unadjusted rate ratio | 0.64 | 0.69 | 0.67 | 0.66 | 0.68 | 0.71 | 0.68 | 0.70 | 0.73 |  |  |
| Adjusted rate ratio  (95% C.I.) | 0.69 (0.66, 0.72) | 0.74 (0.71, 0.77) | 0.72 (0.69, 0.74) | 0.71 (0.69, 0.74) | 0.73 (0.70, 0.76) | 0.75 (0.72, 0.78) | 0.73 (0.70, 0.75) | 0.74 (0.72, 0.77) | 0.77 (0.74, 0.80) |  |  |
| *Hispanic* | 11.7 | 12.7 | 13.2 | 12.2 | 10.9 | 13.4 | 13.8 | 13.0 | 11.0 | < .0001 | 12.4 |
| Unadjusted rate ratio | 0.85 | 0.84 | 0.87 | 0.80 | 0.71 | 0.80 | 0.76 | 0.75 | 0.76 |  |  |
| Adjusted rate ratio  (95% C.I.) | 0.79 (0.74, 0.85) | 0.78 (0.73, 0.84) | 0.82 (0.76, 0.87) | 0.76 (0.71, 0.82) | 0.68 (0.63, 0.73) | 0.76 (0.71, 0.82) | 0.74 (0.69, 0.79) | 0.73 (0.68, 0.79) | 0.76 (0.70, 0.82) |  |  |
| *Asian and North American Native* | 10.1 | 10.9 | 11.5 | 10.7 | 10.8 | 12.3 | 12.8 | 13 | 10 | < .0001 | 11.3 |
| Unadjusted rate ratio | 0.74 | 0.72 | 0.76 | 0.71 | 0.70 | 0.73 | 0.71 | 0.71 | 0.69 |  |  |
| Adjusted rate ratio  (95% C.I.) | 0.77 (0.74, 0.82) | 0.76 (0.72, 0.79) | 0.80 (0.76, 0.84) | 0.74 (0.71, 0.78) | 0.75 (0.72, 0.79) | 0.80 (0.76, 0.83) | 0.78 (0.75, 0.81) | 0.79 (0.76, 0.82) | 0.77 (0.74, 0.80) |  |  |

p-trend = p-value of continuous variable for year. Adjusted for age, gender.

p-value for interaction term between race and year = 0.0035
