# Supplementary material for: Racial and ethnic disparities in diagnosis, management and outcomes of aortic stenosis in the Medicare population
Source: PLoS One. 2023 Apr 10;18(4):e0281811. doi: 10.1371/journal.pone.0281811 (PMC10085041; doi:10.1371/journal.pone.0281811)
Supplement: S4 Table — (DOCX) [file pone.0281811.s004.docx]

**Table S4:** Unadjusted and adjusted prevalence rates and rate ratios

p-trend = p-value of continuous variable for year. Adjusted for age, gender. p-value for interaction term between race and year = 0.0012

| **Characteristic** | **Number of beneficiaries with AS (prevalence rate per 1000 beneficiaries)** | | | | | | | | | | | | | | | | | |  |
| --- | --- | --- | --- | --- | --- | --- | --- | --- | --- | --- | --- | --- | --- | --- | --- | --- | --- | --- | --- |
|  | **2010** | | **2011** | | **2012** | | **2013** | | **2014** | | **2015** | | **2016** | | **2017** | | **2018** | **p-trend** | **2010-2018** |
| **Overall** | 33.5 | | 36.0 | | 37.3 | | 38.3 | | 39.8 | | 42.7 | | 44.6 | | 46.0 | | 40.0 | < .0001 | 39.9 |
| **Age** |  | | | | | | | | | | | | | | | | | | |
| 66-74 | 19.1 | | 19.9 | | 20.5 | | 21.1 | | 21.8 | | 23.7 | | 24.8 | | 26.0 | | 22.0 | < .0001 | 22.1 |
| 75-84 | 38.3 | | 40.8 | | 42.4 | | 43.5 | | 45.6 | | 48.7 | | 51.1 | | 53.0 | | 47.0 | < .0001 | 45.5 |
| 85+ | 55.6 | | 61.9 | | 64.2 | | 66.8 | | 70.8 | | 76.6 | | 81.8 | | 87.0 | | 79.0 | < .0001 | 71.2 |
| **Sex** |  | | | | | | | | | | | | | | | | | | |
| Female | 30.0 | | 32.3 | | 33.6 | | 34.6 | | 35.8 | | 38.3 | | 40.3 | | 42.0 | | 36.0 | < .0001 | 35.9 |
| Male | 38.6 | | 41.1 | | 42.2 | | 43.5 | | 45.3 | | 48.5 | | 50.4 | | 52.0 | | 46.0 | < .0001 | 45.3 |
| **Race/Ethnicity** |  | | | | | | | | | | | | | | | | | | |
| White | 34.7 | | 37.2 | | 38.6 | | 39.9 | | 41.4 | | 44.2 | | 46.3 | | 49.0 | | 42.0 | < .0001 | 41.8 |
| Black | 19.6 | | 22.2 | | 22.4 | | 23.0 | | 24.2 | | 26.5 | | 27.7 | | 30.0 | | 26.0 | < .0001 | 24.9 |
| Hispanic | 26.4 | | 28.0 | | 29.1 | | 28.6 | | 28.0 | | 31.2 | | 32.6 | | 34.0 | | 29.0 | < .0001 | 30.0 |
| Asian and North American Native | 24.0 | | 25.7 | | 27.1 | | 26.5 | | 27.1 | | 29.8 | | 30.9 | | 33.0 | | 28.0 | < .0001 | 28.9 |
|  | | | | | | | | | | | | | | | | | | | |
| **Race/Ethnicity** | **2010** | | **2011** | | **2012** | | **2013** | | **2014** | | **2015** | | **2016** | | **2017** | | **2018** | **p-trend** | **2010-2018** |
| *White (reference)* | 34.7 | | 37.2 | | 38.6 | | 39.9 | | 41.4 | | 44.2 | | 46.3 | | 48.0 | | 42.0 | < .0001 | 41.4 |
| *Black* | 19.6 | | 22.2 | | 22.4 | | 23.0 | | 24.2 | | 26.5 | | 27.7 | | 29.0 | | 26.0 | < .0001 | 24.5 |
| Unadjusted rate ratio | 0.57 | | 0.60 | | 0.58 | | 0.58 | | 0.58 | | 0.60 | | 0.60 | | 0.61 | | 0.61 |  |  |
| Adjusted rate ratio  (95% C.I.) | 0.62 (0.60, 0.63) | | 0.65 (0.63, 0.66) | | 0.63 (0.61, 0.64) | | 0.62 (0.61, 0.64) | | 0.63 (0.62, 0.65) | | 0.65 (0.63, 0.66) | | 0.64 (0.63, 0.66) | | 0.65 (0.64, 0.67) | | 0.66 (0.65, 0.68) |  |  |
| *Hispanic* | 26.4 | | 28.0 | | 29.1 | | 28.6 | | 28.0 | | 31.2 | | 32.6 | | 32 | | 28 | < .0001 | 29.3 |
| Unadjusted rate ratio | 0.76 | | 0.75 | | 0.75 | | 0.72 | | 0.68 | | 0.70 | | 0.70 | | 0.67 | | 0.67 |  |  |
| Adjusted rate ratio  (95% C.I.) | 0.71 (0.67, 0.74) | | 0.70 (0.67, 0.73) | | 0.71 (0.68, 0.74) | | 0.68 (0.65, 0.71) | | 0.65 (0.62, 0.68) | | 0.68 (0.65, 0.71) | | 0.68 (0.65, 0.72) | | 0.66 (0.63, 0.69) | | 0.67 (0.64, 0.70) |  |  |
| *Asian and North American Native* | 24.0 | | 25.7 | | 27.1 | | 26.5 | | 27.1 | | 29.8 | | 30.9 | | 32 | | 28 | < .0001 | 28.2 |
| Unadjusted rate ratio | 0.69 | | 0.69 | | 0.70 | | 0.67 | | 0.66 | | 0.67 | | 0.67 | | 0.67 | | 0.66 |  |  |
| Adjusted rate ratio  (95% C.I.) | 0.73 (0.71, 0.76) | | 0.73 (0.71, 0.75) | | 0.74 (0.72, 0.76) | | 0.71 (0.68, 0.73) | | 0.71 (0.69, 0.73) | | 0.74 (0.72, 0.76) | | 0.75 (0.73, 0.77) | | 0.76 (0.74, 0.78) | | 0.75 (0.73, 0.77) |  |  |
| **Race/Ethnicity: adjusted for gender, age, race*year, dual eligibility** |  | | | | | | | | | | | | | | | | |  |  |
| *White (reference)* | 34.7 | 37.2 | | 38.6 | | 39.9 | | 41.4 | | 44.2 | | 46.3 | | 48.0 | | 42.0 | |  |  |
| *Black - adjusted* | 0.63 (0.62, 0.65) | 0.67 (0.65, 0.68) | | 0.65 (0.63, 0.66) | | 0.64 (0.63, 0.66) | | 0.65 (0.64, 0.67) | | 0.66 (0.65, 0.68) | | 0.66 (0.65, 0.67) | | 0.67 (0.65, 0.68) | | 0.68 (0.66, 0.69) | |  |  |
| *Hispanic - adjusted* | 0.76 (0.72, 0.79) | 0.74 (0.71, 0.78) | | 0.76 (0.72, 0.79) | | 0.73 (0.7, 0.77) | | 0.69 (0.66, 0.73) | | 0.72 (0.69, 0.76) | | 0.73 (0.7, 0.76) | | 0.71 (0.68, 0.74) | | 0.72 (0.68, 0.75) | |  |  |
| *Asian and North American Native - adjusted* | 0.77 (0.74, 0.79) | 0.76 (0.74, 0.79) | | 0.78 (0.75, 0.8) | | 0.74 (0.72, 0.76) | | 0.74 (0.72, 0.76) | | 0.77 (0.75, 0.79) | | 0.78 (0.76, 0.8) | | 0.78 (0.77, 0.8) | | 0.77 (0.75, 0.79) | |  |  |
