# Supplementary material for: Racial and ethnic disparities in diagnosis, management and outcomes of aortic stenosis in the Medicare population
Source: PLoS One. 2023 Apr 10;18(4):e0281811. doi: 10.1371/journal.pone.0281811 (PMC10085041; doi:10.1371/journal.pone.0281811)
Supplement: S5 Table — (DOCX) [file pone.0281811.s005.docx]

**Table S5:** Trends in management of AS (cardiology E&M visits, interventional cardiology E&M visits, cardiothoracic surgery E&M visits, TTE)

|  | **2010** | **2011** | **2012** | **2013** | **2014** | **2015** | **2016** | **2017** | **2018** | **p-trend** |
| --- | --- | --- | --- | --- | --- | --- | --- | --- | --- | --- |
| **Cardiology E&M visit** |  | | | | | | | | | |
| White (per 1K) | 2,088 | 2,010 | 1,898 | 1,848 | 1,760 | 1,728 | 1,674 | 1,579 | 1,639 | < .0001 |
| Black (per 1K) | 1,978 | 1,883 | 1,895 | 1,879 | 1,814 | 1,827 | 1,838 | 1,718 | 1,787 | < .0001 |
| Hispanic (per 1K) | 1,989 | 2,037 | 1,970 | 1,913 | 1,865 | 1,670 | 1,731 | 1,660 | 1,813 | < .0001 |
| Asian and North American Native (per 1K) | 1,698 | 1,678 | 1,705 | 1,633 | 1,595 | 1,551 | 1,610 | 1,530 | 1,604 | < .0001 |
| **Interventional Cardiology E&M visit** |  | | | | | | | | | |
| White (per 1K) | 1,590 | 1,620 | 1,611 | 1,615 | 1,592 | 1,607 | 1,580 | 1,560 | 1,654 | 0.64 |
| Black (per 1K) | 1,584 | 1,609 | 1,661 | 1,671 | 1,598 | 1,631 | 1,639 | 1,649 | 1,765 | 0.68 |
| Hispanic (per 1K) | 1,966 | 1,959 | 1,964 | 1,977 | 1,974 | 1,959 | 1,951 | 1,986 | 1,995 | 0.96 |
| Asian and North American Native (per 1K) | 1,714 | 1,823 | 1,746 | 1,735 | 1,557 | 1,528 | 1,592 | 1,632 | 1,681 | 0.69 |
| **Cardiothoracic Surgery E&M visit** |  | | | | | | | | | |
| White (per 1K) | 121 | 123 | 131 | 139 | 137 | 138 | 140 | 141 | 157 | < .0001 |
| Black (per 1K) | 102 | 107 | 132 | 122 | 126 | 131 | 117 | 127 | 131 | < .0001 |
| Hispanic (per 1K) | 104 | 121 | 120 | 110 | 122 | 121 | 101 | 101 | 191 | < .0001 |
| Asian and North American Native (per 1K) | 90 | 88 | 116 | 112 | 116 | 123 | 133 | 125 | 129 | < .0001 |
| **TTE** |  | | | | | | | | | |
| White (per 1K) | 1,065 | 1,063 | 1,055 | 1,042 | 1,043 | 1,054 | 1,072 | 1,076 | 1,164 | <.0001 |
| Black (per 1K) | 1,180 | 1,184 | 1,178 | 1,148 | 1,156 | 1,145 | 1,171 | 1,163 | 1,233 | <.0001 |
| Hispanic (per 1K) | 1,219 | 1,205 | 1,229 | 1,172 | 1,162 | 1,132 | 1,139 | 1,154 | 1,233 | <.0001 |
| Asian and North American Native (per 1K) | 1,106 | 1,079 | 1,087 | 1,061 | 1,062 | 1,048 | 1,077 | 1,083 | 1,173 | <.0001 |

E&M: Evaluation & management, TTE: transthoracic echocardiography

p-trend = p-value of continuous variable per year
