# Supplementary material for: Racial and ethnic disparities in diagnosis, management and outcomes of aortic stenosis in the Medicare population
Source: PLoS One. 2023 Apr 10;18(4):e0281811. doi: 10.1371/journal.pone.0281811 (PMC10085041; doi:10.1371/journal.pone.0281811)
Supplement: S6 Table — (DOCX) [file pone.0281811.s006.docx]

**Table S6:** Trends in treatment of AS (SAVR, TAVR)

|  | **2010** | **2011** | **2012** | | **2013** | **2014** | **2015** | **2016** | **2017** | **2018** | **p-trend** |
| --- | --- | --- | --- | --- | --- | --- | --- | --- | --- | --- | --- |
| **SAVR** |  | | |  |  |  |  |  |  |  |  |
| White (per 1K) | 37 | 36 | 32 | | 32 | 28 | 26 | 22 | 18 | 19 | < .0001 |
| Black (per 1K) | 27 | 20 | 22 | | 17 | 19 | 17 | 13 | 12 | 10 | < .0001 |
| Hispanic (per 1K) | 31 | 25 | 26 | | 23 | 18 | 22 | 14 | 11 | 13 | < .0001 |
| Asian and North American Native (per 1K) | 19 | 22 | 21 | | 20 | 23 | 19 | 22 | 20 | 18 | < .0001 |
| **TAVR (2012-2016)** |  | | |  |  |  |  |  |  |  |  |
| White (per 1K) | 0 | 1 | 5 | | 9 | 13 | 18 | 23 | 28 | 36 | < .0001 |
| Black (per 1K) | 0 | 1 | 4 | | 6 | 8 | 12 | 14 | 18 | 20 | < .0001 |
| Hispanic (per 1K) | 0 | 0 | 4 | | 5 | 11 | 17 | 12 | 22 | 27 | < .0001 |
| Asian and North American Native (per 1K) | 0 | 0 | 4 | | 4 | 8 | 10 | 18 | 16 | 22 | < .0001 |

SAVR: surgical aortic valve replacement, TAVR: transcatheter aortic valve replacement

p-trend = p-value of continuous variable per year

(Un)adjusted TAVR rates were calculated beginning in 2012 to account for the first full year of TAVR.
