# Supplementary material for: Racial and ethnic disparities in diagnosis, management and outcomes of aortic stenosis in the Medicare population
Source: PLoS One. 2023 Apr 10;18(4):e0281811. doi: 10.1371/journal.pone.0281811 (PMC10085041; doi:10.1371/journal.pone.0281811)
Supplement: S7 Table — (DOCX) [file pone.0281811.s007.docx]

**Table S7:** Trends in AS outcomes (all-cause hospitalizations, heart-failure hospitalizations, 1-year mortality

|  | **2010** | **2011** | **2012** | | **2013** | **2014** | **2015** | **2016** | **2017** | **2018** | **p-trend** |
| --- | --- | --- | --- | --- | --- | --- | --- | --- | --- | --- | --- |
| **All-cause hospitalization** |  | | |  |  |  |  |  |  |  |  |
| White (per 1K) | 666 | 662 | 644 | | 625 | 647 | 637 | 650 | 641 | 654 | < .0001 |
| Black (per 1K) | 828 | 898 | 838 | | 770 | 823 | 788 | 821 | 811 | 834 | < .0001 |
| Hispanic (per 1K) | 759 | 816 | 720 | | 760 | 725 | 728 | 736 | 722 | 748 | < .0001 |
| Asian and North American Native (per 1K) | 526 | 570 | 539 | | 497 | 523 | 526 | 528 | 507 | 541 | < .0001 |
| **Heart failure hospitalization** |  | | |  |  |  |  |  |  |  |  |
| White (per 1K) | 275 | 299 | 301 | | 297 | 316 | 318 | 337 | 339 | 358 | < .0001 |
| Black (per 1K) | 392 | 465 | 453 | | 427 | 458 | 447 | 488 | 483 | 524 | < .0001 |
| Hispanic (per 1K) | 323 | 381 | 348 | | 392 | 349 | 368 | 393 | 416 | 449 | < .0001 |
| Asian and North American Native (per 1K) | 202 | 260 | 239 | | 232 | 247 | 256 | 266 | 266 | 293 | < .0001 |
| **1-year mortality** |  | | |  |  |  |  |  |  |  |  |
| White (per 1K) | 102 | 108 | 110 | | 107 | 108 | 106 | 107 | 104 | 110 | 0.09 |
| Black (per 1K) | 99 | 122 | 118 | | 109 | 118 | 113 | 114 | 112 | 116 | 0.08 |
| Hispanic (per 1K) | 96 | 119 | 110 | | 123 | 118 | 105 | 118 | 115 | 119 | 0.08 |
| Asian and North American Native (per 1K) | 63 | 84 | 85 | | 82 | 87 | 72 | 80 | 72 | 81 | 0.12 |

p-trend = p-value of continuous variable per year
